# Identification of Cofilin‐1 as a novel biomarker of atopic dermatitis using iTRAQ quantitative proteomics

**DOI:** 10.1002/jcla.24751

**Published:** 2022-10-28

**Authors:** Xiaotao Zhou, Bo Xiao, Jiajia Zeng, Liying Zhou, Xiaodong Wang, Shangqi Zhao, Xiaobo Li, Huiqiu Zhang, Yanjun Su, Zhenyu Zhao, Xichuan Li

**Affiliations:** ^1^ Department of Immunology, School of Basic Medical Sciences Xinjiang Medical University Urumqi Xinjiang China; ^2^ Department of Immunology, School of Basic Medical Sciences Tianjin Medical University Tianjin China; ^3^ Research and Development Center Beijing Tide Pharmaceutical Co., Ltd Beijing China; ^4^ Department of Dermatology First Affiliated Hospital of Xinjiang Medical University Urumqi Xinjiang China; ^5^ Tianjin Key Laboratory of Animal and Plant Resistance, College of Life Sciences Tianjin Normal University Tianjin China; ^6^ Department of Lung Cancer, Key Laboratory of Cancer Prevention and Therapy, Tianjin Lung Cancer Center, National Clinical Research Center for Cancer, Tianjin's Clinical Research Center for Cancer Tianjin Medical University Cancer Institute and Hospital Tianjin China; ^7^ Departments of Pharmacy, NHC Key Laboratory of Hormones and Development, Tianjin Key Laboratory of Metabolic Diseases Tianjin Medical University Chu Hsien‐I Memorial Hospital Tianjin China

**Keywords:** atopic dermatitis, biomarker, Cofilin‐1, iTRAQ, skin barrier

## Abstract

**Background:**

Atopic dermatitis (AD) is a chronic relapsing inflammatory skin condition; however, little is known about the pathogenesis and serum biomarker of this disease.

**Methods:**

Isobaric tagging for relative and absolute quantitation (iTRAQ) proteomic assay was adopted to identify and quantify the differentially expressed proteins (DEPs) in the serum of AD patients. Bioinformatic analysis, including GO, Reactome, GSEA, PPI, and ssGSEA analysis, were used to identified the enriched pathways, hub proteins and immune cells. The expression level and distribution of hub proteins were confirmed by ELISA and IHC.

**Results:**

Sixty‐six DEPs were identified with iTRAQ proteomic assay by analyzing serum from AD patients and normal subjects. GO and Reactome analysis shown the alternated pathway were mainly involved in immunity, oxidative stress, and actin cytoskeleton. The GSEA and PPI network analysis among the DEPs were carried out and identified Cofilin‐1 and profilin‐1 as the core components of this network. Additionally, the disruption of Th1/Th2/Th17 cell balance and the significantly reducing of Treg, MDSC, and γδT cells was also found in AD patients using the ssGSEA analysis. Further ELISA and IHC assay validated the significantly elevated expression of Cofilin‐1 in AD patients.

**Conclusion:**

Our results suggested that Cofilin‐1 may serve as a novel biomarker for AD diagnosis.

## Graphical abstract

To identify novel biomarkers of AD, the proteomes of serum samples from 15 AD patients and 15 healthy individuals were quantitatively profiled using the iTRAQ reagent with LC–MS/MS analysis. Based on the protein quantitative standard (the fold change [FC] > 1.5; probability [*p*] value <0.05), a total of 66 proteins displayed significant differential expression levels between AD patients and healthy normal controls. Here is the workflow of proteomic analysis applied in this study.

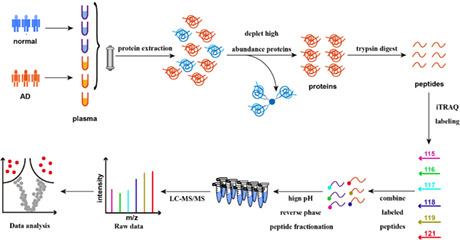

## 1 INTRODUCTION

Atopic dermatitis (AD) is a chronic inflammatory skin disease that is characterized by intense itching and recurrent eczematous lesions.^1^ Approximately 70% of AD cases started in children under 5 years of age, albeit other cases were reported from patients at any age.^2^ This disease dramatically plagued both patients and their families. Although the pathogenesis of AD remained unclear, current research indicated that disorder in the immune system and damage of skin barrier both play inevitable roles in the occurrence and development of AD.^3^, ^4^ What is more, certain studies showed that other factors affected the integrity of the epithelial barrier, such as the polymerization of actin,^5^ the abnormal level of factors or plasma proteins,^6^, ^7^ pathological cell adhesion^8^ and skin adhesion caused by bacteria infection,^2^, ^9^ were closely related with the occurrence of AD.

The diagnosis of AD relies exclusively on clinical features, because of no specific laboratory or histological biomarkers have been clinical reported. Several proteomic studies using skin tissue or serum from AD patients have been performed to help identify specific biomarkers of AD recently,^6^, ^10^, ^11^, ^12^, ^13^ but so far there are not enough sensitive and specific biomarkers has been applied for clinical diagnosis, let alone enough biomarkers for the assessment of severity or treatment effects. Therefore, more quantitative proteomic studies are still needed. Our research adopted isobaric tagging for relative and absolute quantitation (iTRAQ) labeling strategy to identify serum biomarkers of AD. iTRAQ was a widely used markup strategy in identifying the differential expression of proteins from complex sample. Due to advantages in accuracy and reliability over traditional 2DE proteomics analysis,^14^ the proteomics technology based on iTRAQ currently became a powerful tool in understanding the pathogenesis of diseases.

In this report, 66 differentially expressed proteins (DEPs) were identified with iTRAQ quantitative proteomic assay technology by analyzing serum from AD patients and normal subjects. These DEPs are mainly involved in immunity, oxidative stress and actin cytoskeleton, which were discovered by GO and Reactome pathway analyses. Furthermore, TLN1, CFL1, TPM3, PFN1, COTL1, and TAGLN2 were identified as latent hub proteins by GSEA and PPI analysis. Then we use the ssGSEA analysis to quantify the level of immune cell infiltration in the AD patients. The differential expression of Cofilin‐1 and Profilin‐1 in the AD patients' serum were confirmed by ELISA. The distribution of Cofilin‐1 in the AD patients' lesional skin were performed by IHC. Our report suggest that Cofilin‐1 could be considered as a potential diagnosis biomarker of AD.

## 2 MATERIALS AND METHODS

### 2.1 The screening and sample collection of AD patients

Samples from AD patients and healthy controls in this study were collected from the First Affiliated Hospital of Xinjiang Medical University. The focal area of suspected pediatric patients was diagnosed firstly according to current diagnostic criterion of AD including dry skin, wrinkles, eczema, pimples, etc. To exclude the other influence factors, patients with medical history, such as asthma, food allergies, and skin inflammation or allergic caused by exogenous stimulation, were ruled out after survey. The diagnosis of patients was further confirmed with the level of total IgE and sIgE in the blood and other diagnostic methods. A total of 90 subjects were enrolled in this study, which including 45 AD patients and 45 healthy controls. Fifteen AD patients and 15 healthy controls were chosen at random for proteomic studies, while the rest were used for ELISA detection. The clinical characteristics of the 90 subjects are shown in Table 1, there were no significant differences in gender and age between the two groups. Blood samples from these subjects were collected and centrifuged at 3000 *g* at 4°C for 20 min in 1 h, and the serum samples were collected and frozen immediately at −80°C. This study was approved by the Regional Research Ethics Committee and was conducted according to the Declaration of Helsinki principles.

**TABLE 1:** The clinical characteristics of healthy controls (HC) and AD patients (AD).

Characteristics	HC (*n* = 45)	AD (*n* = 45)	*p*‐Value
Gender			
Female	21	22	0.833
Male	24	23	
Age (years)			
<14	30	34	0.352
≥14	15	11	
Disease duration (months)			
<12	—	14	–
≥12	—	31	

### 2.2 iTRAQ sample preparation, labeling and LC–MS/MS analysis

Equal volume of serum samples from five patients in each group were pooled for iTRAQ analysis. The multiple affinity removal LC column – human 14 (Agilent Technologies) was used to remove the high abundance proteins from the samples, accomplishing by ultrafiltration and concentration in the 10 kDa ultrafiltration tubes. Peptide mixtures (100 μg) from each group were labeled using the iTRAQ Reagent‐plex Multiplex Kit (AB SCIEX) following manufacturer's protocol. The digested peptides were labeled with six iTRAQ tags (reagent 115:normal 1; reagent 118:AD1; reagent 116:normal 2; reagent 119:AD 2; reagent 117:normal 3; reagent 121:AD 3). The labeled peptides of each group were mixed and graded by Agilent 1260 infinity II HPLC system. Then liquid chromatography‐mass spectrometry/mass spectrometry (LC–MS/MS) analysis was performed on Easy nLC (Thermo Scientific).

### 2.3 Data analysis of iTRAQ experiments and bioinformatics analysis

The original data of iTRAQ assay were collected by mass spectrometry, and the search of the database was conducted by the MaxQuant software (version 1.6.1.0). Reporter ions based on MS2 was selected as the quantitative type, N‐terminal and Lys were selected as the marker sites for each mass tag, the enzyme cut style of Trypsin/P, variable modification of Oxidation (M), Acetyl (protein N‐term), fixed modification of Carbamidomethyl (C), the primary quality deviation of 20 ppm, and the secondary quality deviation of 0.05 Da. The database is UniProt‐Swiss human database (including 20,443 annotated proteins, released 2019.07). The minimum peptide length was six amino acids, the maximum peptide mass was 5000 Da, the FDR of spectrum and protein matching was set to 1% and quantitative value of protein was obtained by iBAQ algorithm. Log 2 transformation of the original quantitative value is carried out to make it to meet the normal distribution. The proteins without missing value were screened for statistical analysis. Based on *p* < 0.05 and FC > 1.5, the differential expression proteins (DEPs) was screened by the *t*‐test function of R language base software package. The cluster profiler software package was used for enrichment analysis of GO and Reactome Pathway of DEPs and the gene set enrichment analysis (GSEA) of all proteins with gene set database of c5.all.v6.2.symbols.gmt of the Molecular Signatures Database (MSigDB) and the adjusted *p* < 0.05. STRING (https://string‐db.org/, version 11.0) was applied to analyze the protein–protein interactions (PPIs) of identified DEPs.

### 2.4 ELISA

The concentrations of Cofilin‐1 and Profilin‐1 in serum from AD patients and healthy controls were quantified by ELISA according to the manufacturer's instructions. All assays were performed in triplicate.

### 2.5 IHC analysis

We performed immunohistochemistry staining for Cofilin‐1 on AD lesion skin and normal skin. The samples were taken from the skin lesion that was most severe. The biopsy specimens were taken about 2 cm in diameter, deeply enough to reach subcutaneous tissue. Briefly, 5 μm thick tissue sections from formaldehyde‐fixed and paraffin‐embedded samples were dewaxed and rehydrated. Rabbit anti‐human CFL1 antibody (ab42824; Abcam) was applied, followed by SP staining. IHC assay were performed as described previously.^15^

### 2.6 Statistical analyses

Statistical analyses were performed with SPSS Statistics 20.0 (SPSS Inc.) and GraphPad Prism 6.0 software. The data were reported as the means ± SD. Quantitative variables were analyzed by Student's *t* tests, and *p* < 0.05 was considered significant.

## 3 RESULTS

### 3.1 Comparative analysis of serum proteomic changes in patients with AD and normal

**FIGURE 1:**
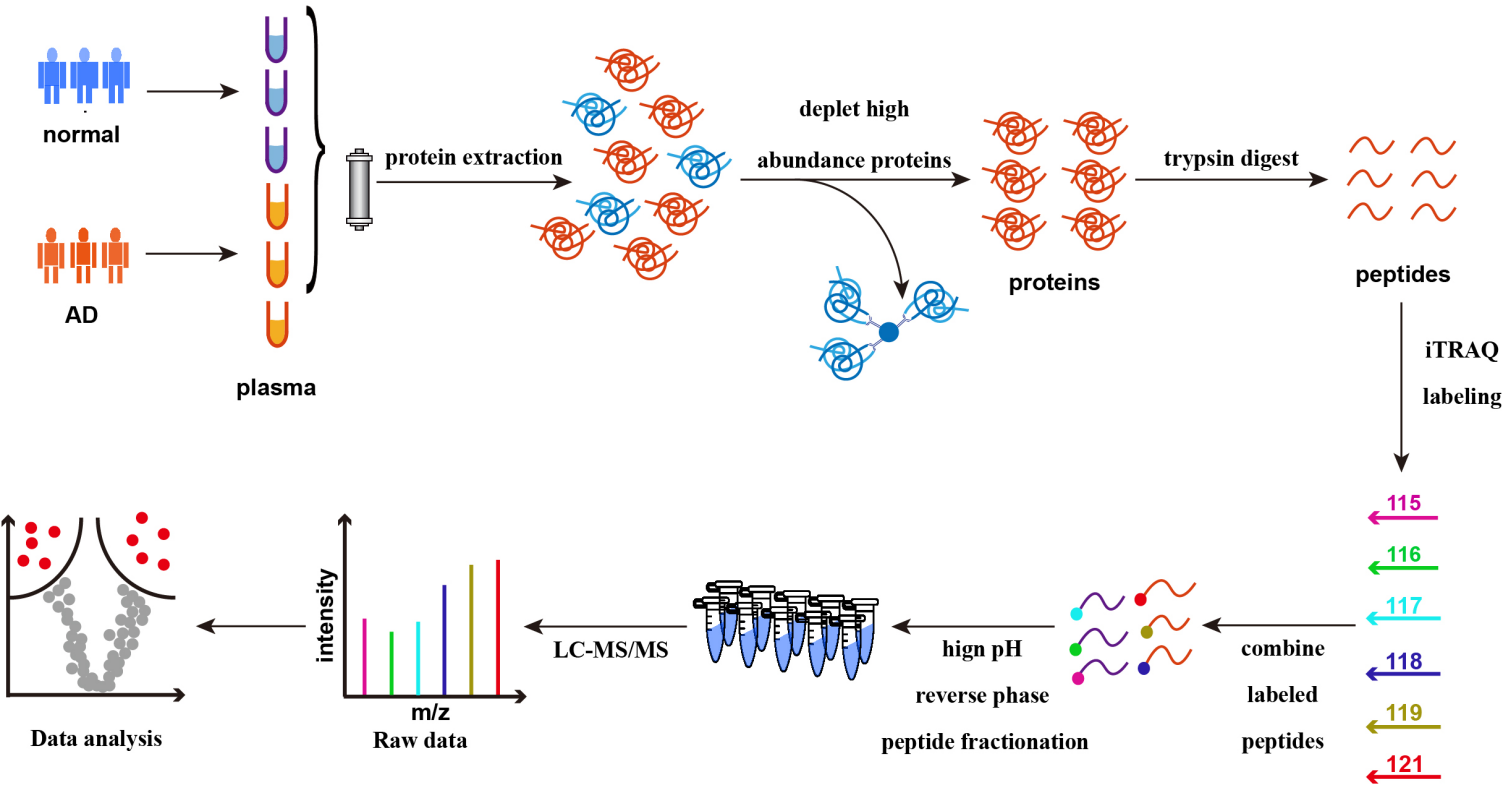
The workflow of proteomic analysis applied in this study. iTRAQ labeling was employed in serum sample preparation.

**FIGURE 2:**
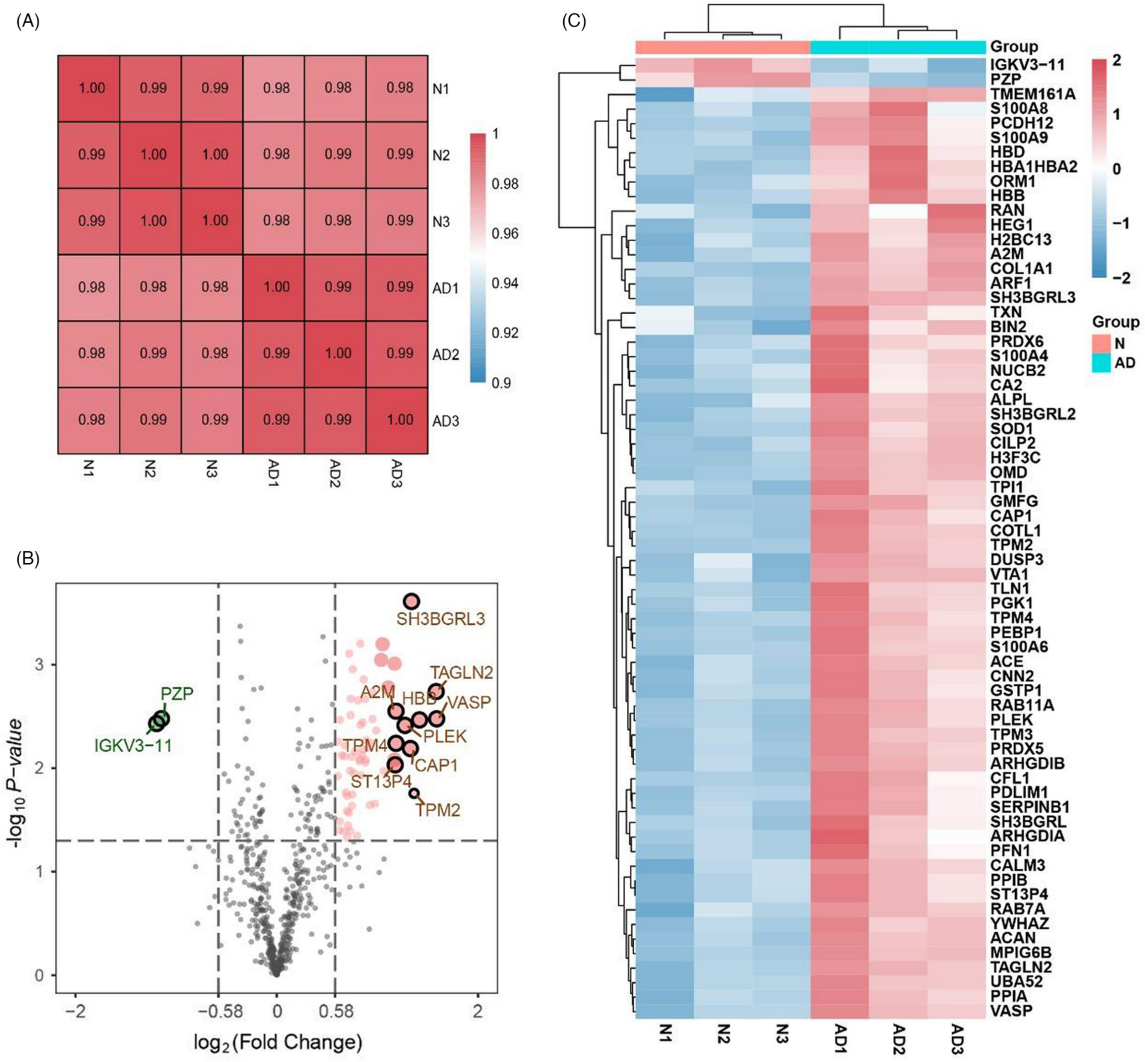
Differential expression of proteins in children with AD. (A) Pearson correlation coefficient between different samples. The *X* and *Y* axes are the intensity of proteins. (B) Volcano plot displays proteins with statistical significance. The top 10 proteins, with the largest fold change, of upregulated and downregulated were labeled. (C) All differentially expressed proteins were showed with heatmap. AD, atopic dermatitis group; N, healthy normal group.

To identify novel biomarkers of AD, the proteomes of serum samples from 15 AD patients and 15 healthy individuals were quantitatively profiled using the iTRAQ reagent with LC–MS/MS analysis. As showed in Figure 1, proteins were digested into peptides by the trypsin buffer, peptides were labeled with six iTRAQ tags and 100 ug iTRAQ‐labing peptides were finally separated by LC–MS/MS analysis. The Pearson correlation coefficient conducted on quantitative data showed that the correlation coefficient between the samples within and between groups is all above 0.97 (Figure 2A), which represents the high repeatability and reliability of data. Based on the protein quantitative standard (the fold change [FC] > 1.5; probability [*p*] value <0.05), a total of 66 proteins displayed significant differential expression levels between AD patients and healthy controls. Figure 2B,C displays the 64 upregulated and the 2 downregulated proteins in AD patients.

### 3.2 GO and Reactome pathway analyses of differentially expressed proteins

**FIGURE 3:**
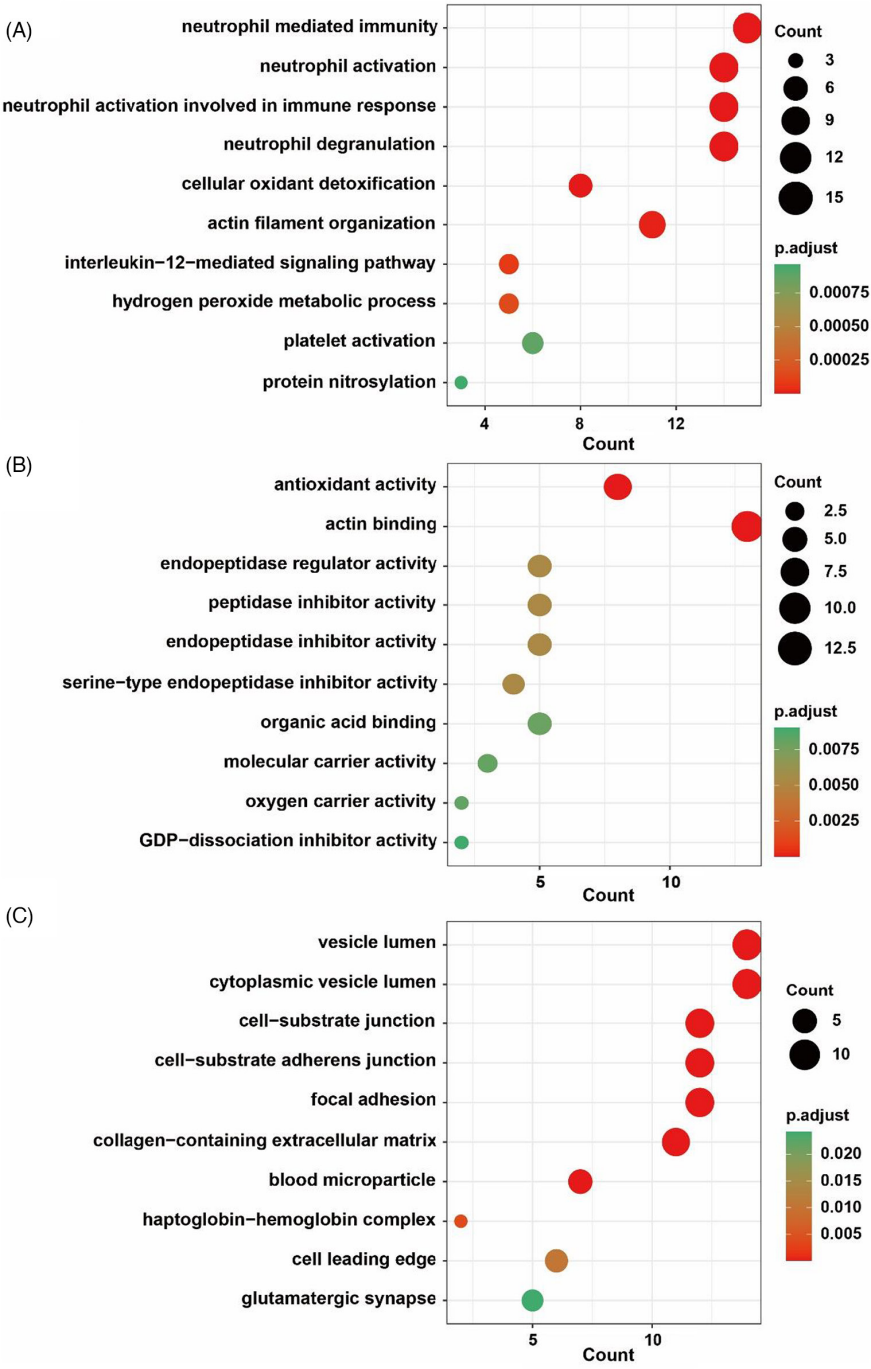
The gene‐ontology enrichment analysis of differentially expressed proteins in atopic dermatitis. (A) Biological process (BP); (B) molecular function (MF); (C) cellular component (CC).

The gene‐ontology (GO) enrichment analysis, which including the biological process (BP), molecular function (MF), cellular component (CC) of gene products, was used for analysis of 66 DEPs through the R package cluster Profiler. Based on the Fisher's Exact Test, remarkable discrepancy contains 36BPs, 16MFs and 10CCs between patients and control groups was observed and the top 10 categories, with the minimum adjusted *p*‐value, were showed as Figure 3. The result shows that these DEPs are mainly involved in immunity, oxidative stress and actin cytoskeleton. As to immunity, neutrophil mediated immunity (15 proteins in 61 DEPs), neutrophil degranulation (14/61), neutrophil activation involved in immune response (14/61) and neutrophil activation (14/61) relating BP were the most abundant. Considering the actin cytoskeleton, actin filament organization (11/61) relating BP, actin binding (13/60) relating MF, cell‐substrate junction (14/62) and cell‐substrate adherens junction (14/62) relating CC were the most prevalent.

**FIGURE 4:**
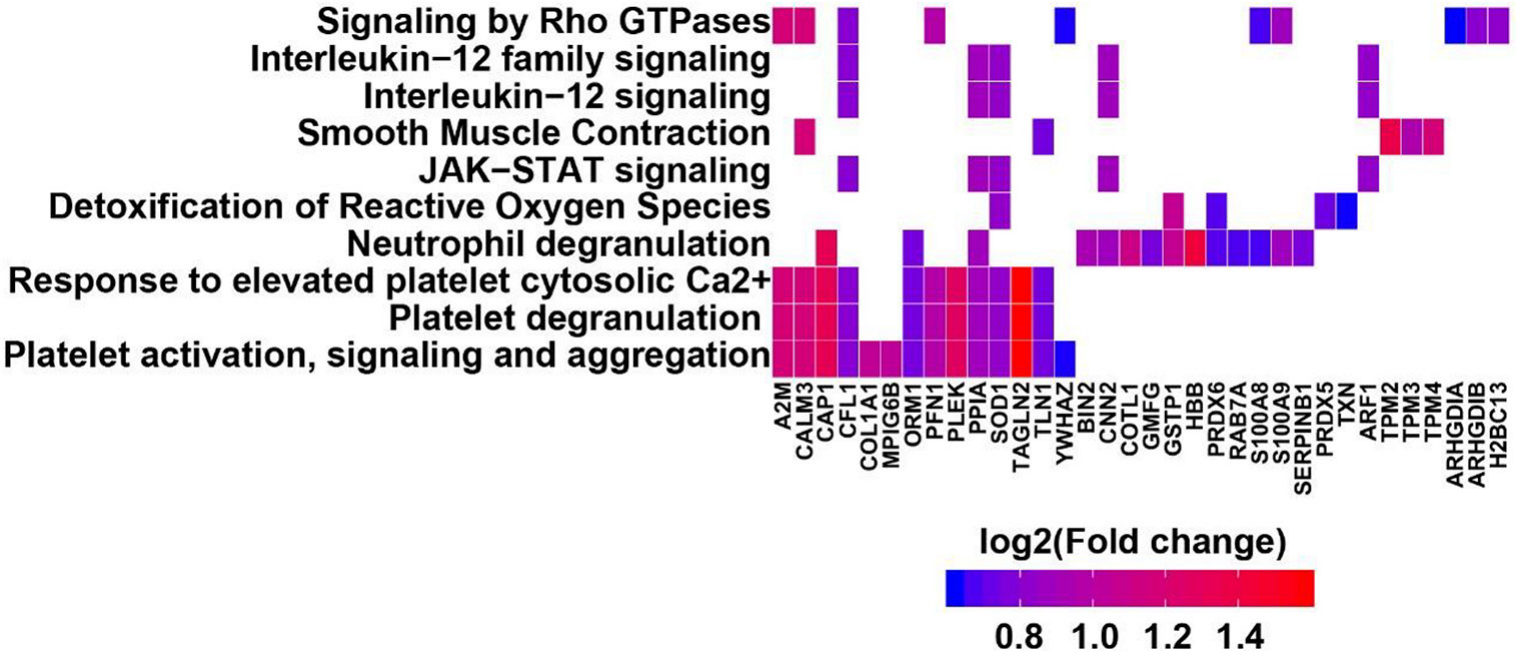
The Reactome pathway analysis of differentially expressed proteins in atopic dermatitis.

Besides, the Reactome pathway analysis has been used to determine the significantly enrichment pathways for DEPs compared with the entire genome background. The 66 DEPs were allocated to 22 Reactome pathways, which show significant enrichment in immune response and actin cytoskeleton, such as neutrophil degranulation, interleukin‐12 signaling and signaling by Rho GTPases, deviation came to 28.57%, 10.20%, and 18.37%, respectively. The top 10 pathways, with the minimum adjusted *p*‐value, and related genes were shown in Figure 4. Collectively, besides direct immunology disorder, the findings provide relevant information to guide future research on the molecular basis of the role of proteins associated with actin cytoskeleton in AD process.

### 3.3 Gene set enrichment analysis and PPI networks of differentially expressed proteins

**FIGURE 5:**
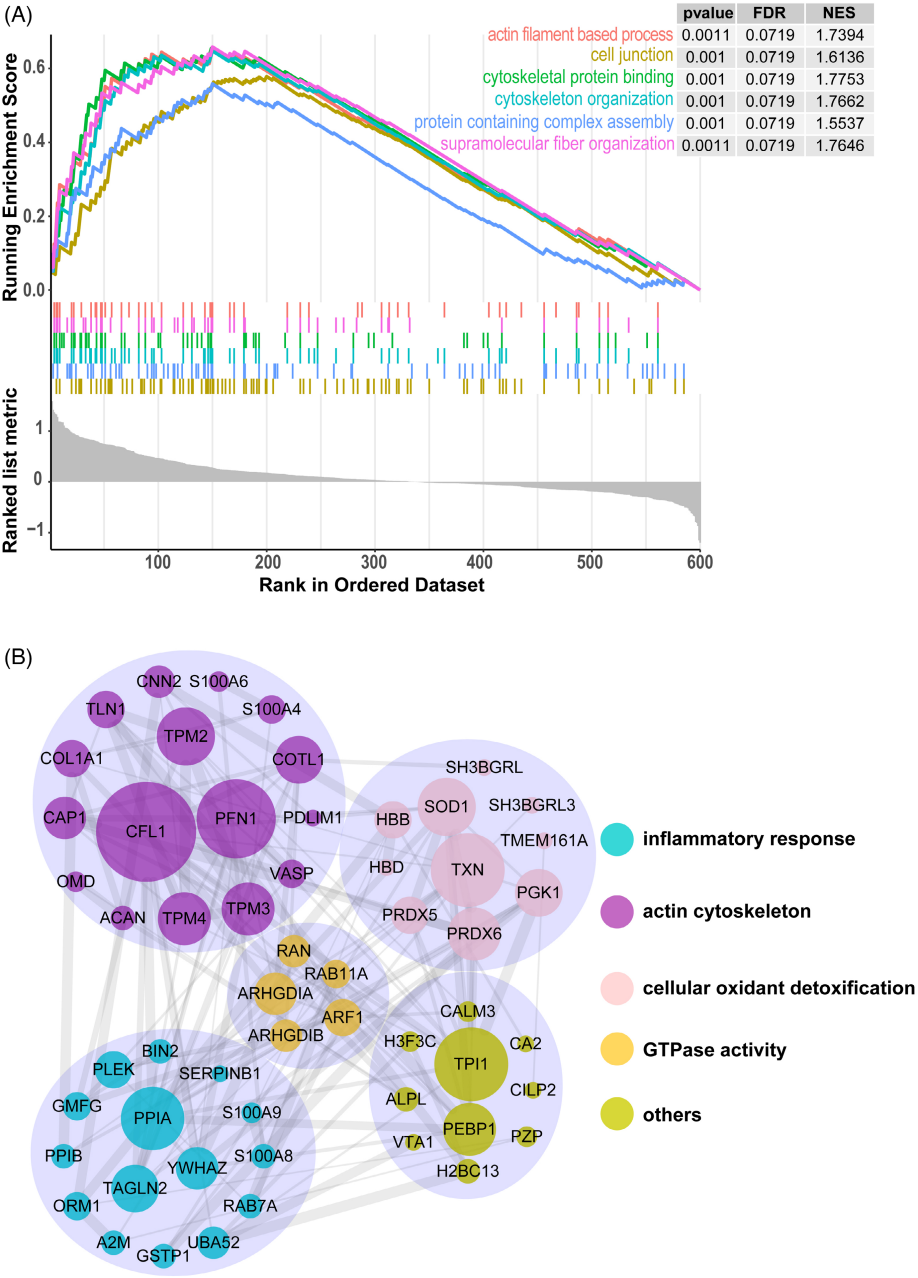
Gene set enrichment analysis and protein–protein interaction analysis of proteins expression. (A) Gene set enrichment analysis of actin cytoskeleton related proteins. (B) Proteins and their interactions were displayed as nodes and edges, and proteins without any interactions were not shown. The interacted proteins were clustered based on their known biological or molecular functions.

Gene set enrichment analysis including GO analysis and pathway enrichment analysis is a tool for threshold‐free enrichment analysis of sequencing data. Recognizes as the most effective way to identify predefined gene sets that demonstrate differential expression levels in normal and abnormal samples, GSEA is used to analyze the DEPs with quantitative value. Figure 5A showed the GSEA analysis of actin filament‐based process, cell junction, cytoskeleton protein binding, cytoskeleton organization, protein containing complex assembly and supermolecule fiber organization, results showing that many gene sets related to actin cytoskeleton were significantly enriched and upregulated, which in line with the GO and Reactome analyses.

Further PPI assays based on DEPs deepen the understanding of protein structure and function using the String database. The result demonstrated that inflammatory response, actin cytoskeleton, cellular oxidant detoxification and GTPase activity played critical roles in the AD pathologic process, with PPIA, CFL1, TXN and ARHGDIA identified as latent hub protein, respectively (Figure 5B).

### 3.4 Immune cell infiltration analysis by single‐sample gene set enrichment analysis

**FIGURE 6:**
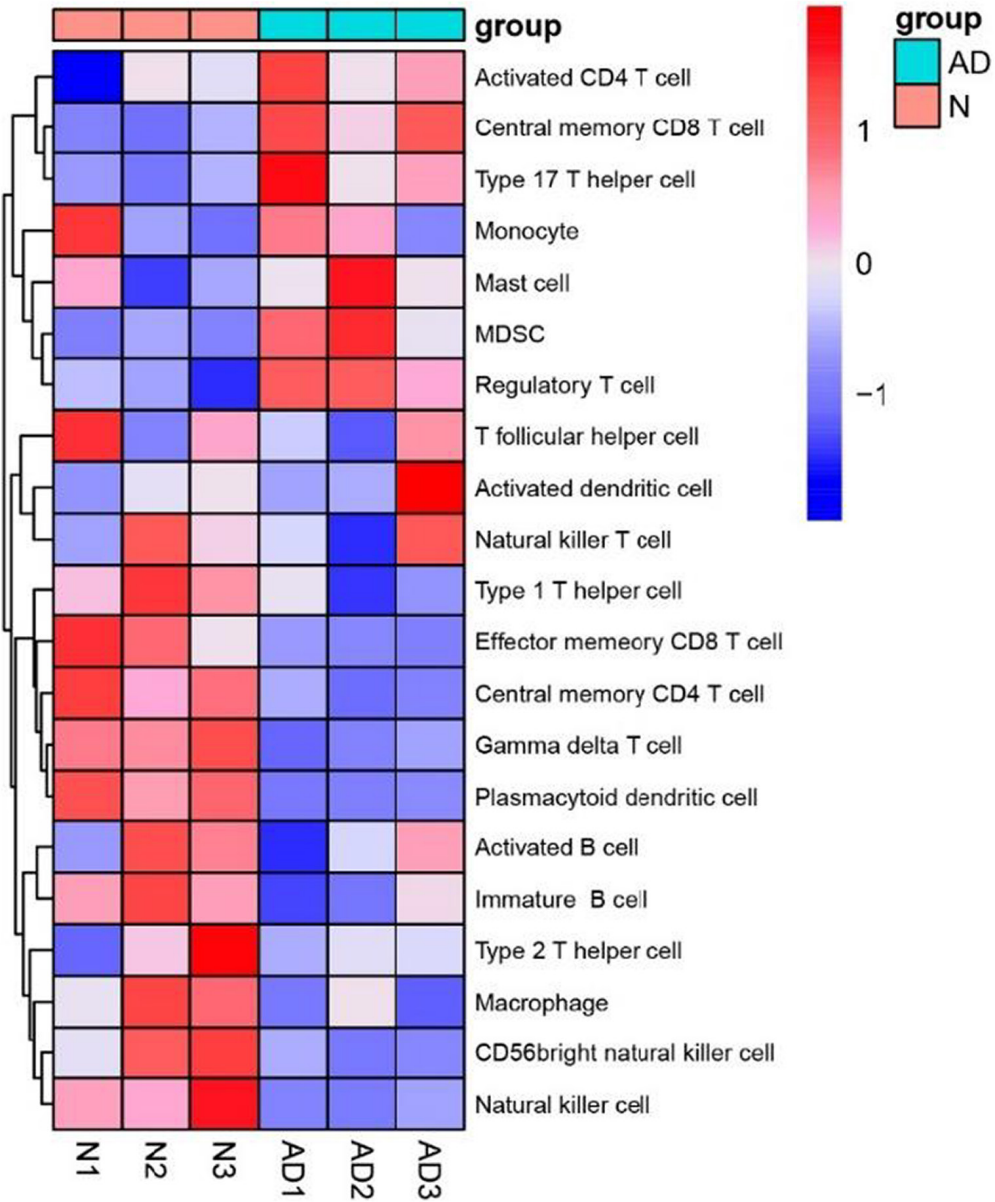
Immune infiltration analysis showed that the infiltration level of various immune cells in children with atopic dermatitis were changed. The infiltration levels of immune cells were quantified by the method of single‐sample gene set enrichment analysis in R package gsva.

The distribution of various immune cells in samples can be obtained by specific bioinformatics software analysis, so as to identify the infiltrated immune cell subsets, compare the expression of different subsets, and further screen a certain immune cell subset as biomarker. In view of the above results repeatedly suggesting the influence of immune factors on AD, we use the single‐sample gene set enrichment analysis (ssGSEA) to quantify the level of immune cell infiltration in the AD patients to find the latent immune mechanism, the results were shown in Figure 6. The activated CD4^+^ T cells, central memory CD8^+^ T cells, Th17 cells, MDSCs, and Treg cells were significantly enriched in AD patient's serum, while the central memory CD4^+^ T cells, effector memory CD8^+^ T cells, Th1 cells, Th2 cells, γδT cells, macrophages, and NK cells were significantly reduced in AD patient's serum. In addition to the widely reported disruption of Th1/Th2/Th17 balance in AD patients, abnormal distribution of Treg, MDSC, and γδT cells was also found.

### 3.5 Verification of actin cytoskeleton gene expression in serum of AD patients

**FIGURE 7:**
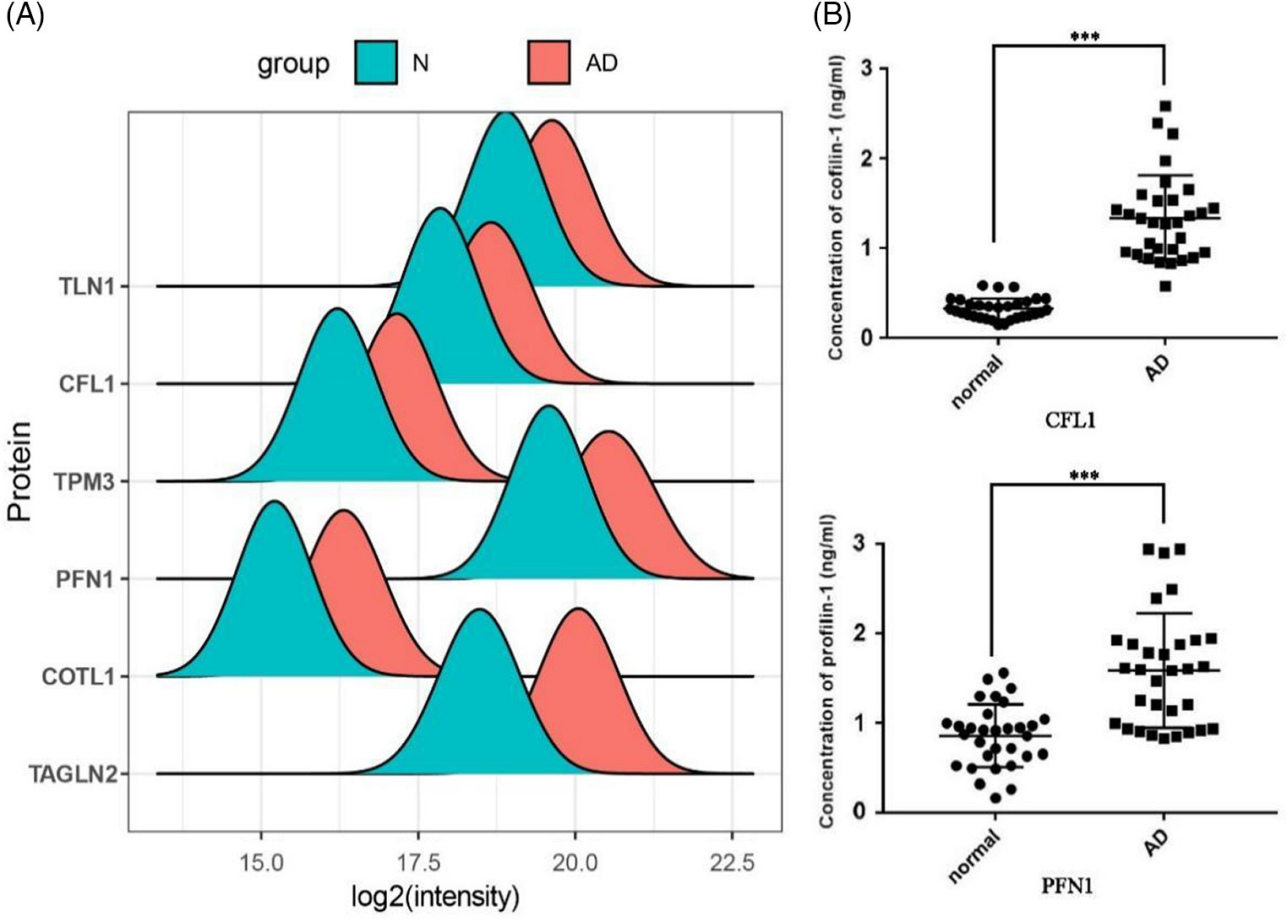
The expression level change of actin cytoskeleton hub genes. (A) The expression level change of CFL1, TLN1, TPM3, PFN1, COTL1 and TAGLN2 identified by GO and GSEA analysis; (B) the expression level of CFL1/Cofilin‐1 and PFN1/Profilin‐1 detected by ELISA in AD patients compared with normal group. ***p < 0.001 (AD serum vs. normal serum). AD, atopic dermatitis; GO, gene‐ontology; GSEA, gene set enrichment analysis.

According to the results of GO and GSEA analysis, the expression of several actin cytoskeleton hub genes were significantly upregulated, including TLN1, CFL1, TPM3, PFN1, COTL1 and TAGLN2 (Figure 7A). Previous research found that CFL1/Cofilin‐1 expression was significantly increased in AD patients and living skin–equivalent (LSE) models, which were caused by mutations or downregulation of filaggrin, a key epidermal barrier protein.^16^ PFN1/Profilin‐1 was also markedly expressed in the skin and serum of patients with psoriasis, another chronic inflammatory skin disease, and could be used as a biomarker and therapeutic target of psoriasis.^17^ To validate the expression level of these two most likely candidate proteins, serum derived from another 30 AD patients and 30 healthy individuals were determined by ELISA. The average expression levels of Cofilin‐1 and Profilin‐1 in the serum of the healthy normal group were 0.32 ng/ml and 0.86 ng, respectively; which were 1.33 ng/ml and 1.59 ng/ml in the serum of AD patients, respectively (Figure 7B). The level of Cofilin‐1 and Profilin‐1 were both upregulated in the serum of the AD group compared with the healthy normal group, which was in accordance with the result of iTRAQ. These ELISA results supporting the conclusion draw from the result of iTRAQ.

### 3.6 High expression of Cofilin‐1 in skin injury tissue of AD patients

**FIGURE 8:**
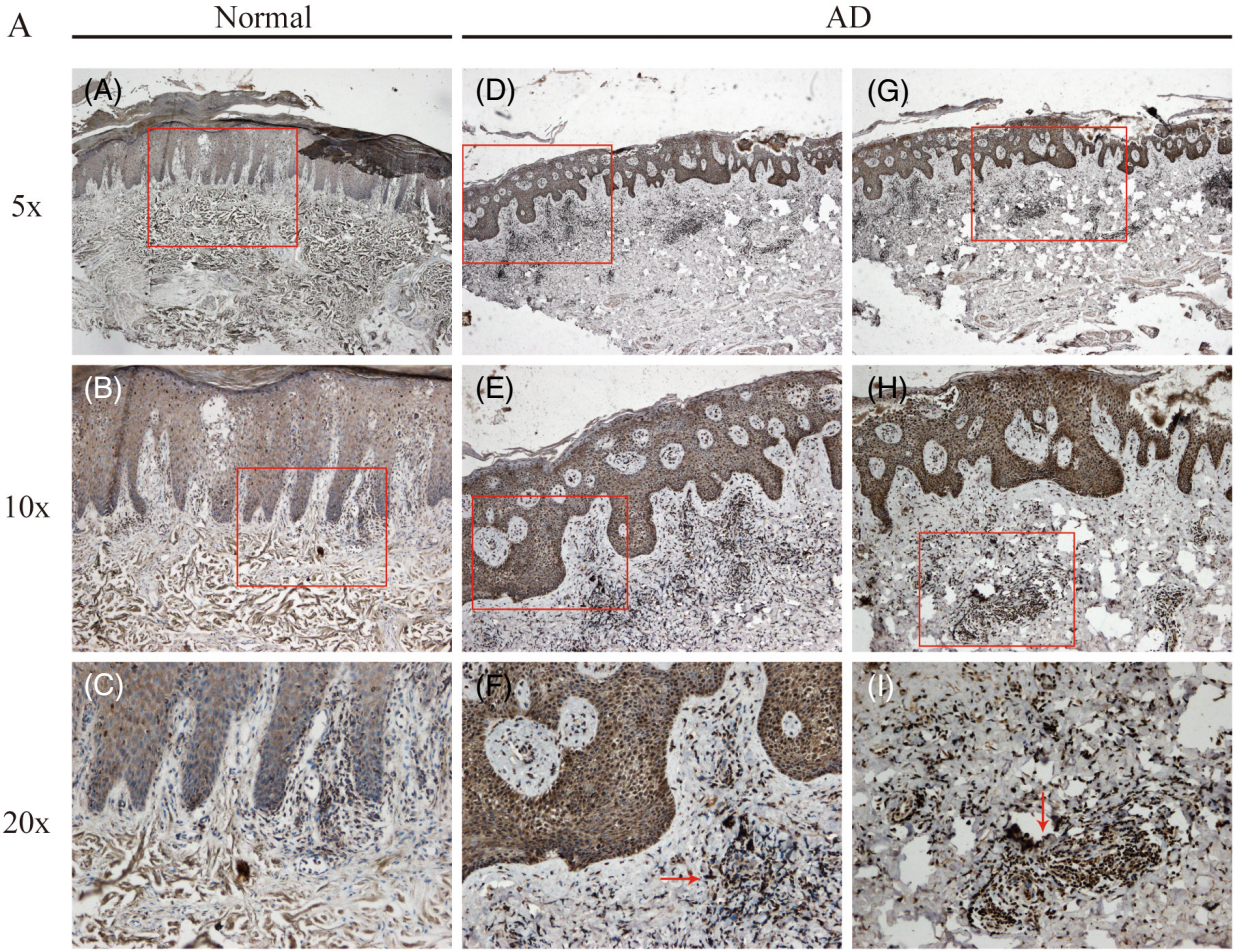
Cofilin‐1 is highly expressed in skin injury tissue of AD patients. IHC staining with anti‐Cofilin‐1 was performed on healthy controls (A–C) and skin injury tissues of AD patients (D–I). AD, atopic dermatitis.

CFL1/Cofilin‐1 is a widely distributed intracellular actin‐modulating protein that binds and depolymerizes filamentous F‐actin and inhibits the polymerization of monomeric G‐actin in a pH‐dependent manner.^18^, ^19^ Increased expression and phosphorylation of Cofilin‐1 by LIM kinase aids in Rho‐induced reorganization of the actin cytoskeleton.^20^ To further investigate the function of Cofilin‐1 in the progress of AD, IHC analysis was used to analyze the distribution and expression level in skin injury tissues of AD patients and healthy controls. The result highlighted that Cofilin‐1 was highly expressed in inflammatory cells of injured skin in AD patients, and moderately expressed in sebaceous glands and epidermal cells (Figure 8). In addition, the expression and distribution of PFN1 were also measured by IHC, but they did not differ significantly between controls and skin injury tissue from AD patients (data not shown). Based on these results, Cofilin‐1 may be involved in skin damage and immune infiltration during AD progress, and could be conclusively considered as a novel biomarker for AD diagnosis.

## 4 DISCUSSION

AD is a chronic relapsing skin disease whose pathomechanisms remains unknown. Possible causes of AD can be involved in genetics, immunological disorders, altered skin barrier,^1^, ^2^ etc. Among those causes, the alternation of skin barrier is worth to be noted because it major contributes to the deterioration of the disease. The dysfunction of skin barrier would make skin more sensitive to infection, bacteria invasion and environmental stimuli.^21^ Additionally, remedy of the damaged skin barrier can serve as potential medical target for AD. Proteins involved in skin barrier may be identified as a diagnosis biomarker of AD.^22^

Proteomic studies have been used to investigate the DEPs and alternated pathways in AD and other skin diseases.^10^, ^11^, ^12^, ^13^ Our research utilized iTRAQ quantitative proteomic technology identified the differentially expressed proteins in AD patients. These DEPs are mainly involved in immunity, oxidative stress and actin cytoskeleton, which were discovered by GO and Reactome pathway analyses. Furthermore, GSEA and PPI analysis also demonstrated inflammatory response, actin cytoskeleton, cellular oxidant detoxification and GTPase activity played critical roles in the AD pathologic process, with PPIA, CFL1, TXN and ARHGDIA identified as latent hub protein, respectively.

Cofilin‐1, encoded by CFL1 gene, was at the core of the protein network and was upregulated in AD patients. Cofilin‐1 is a pH‐dependent actin‐modulating protein, which will depolymerize actin filaments under elevated pH and is crucial for cell mobility.^23^ It was also reported that Cofilin‐1 was involved in the invasion,^24^, ^25^ differentiation^26^, ^27^ and epithelial‐mesenchymal transition of cancer cell,^28^, ^29^ which highlight the importance of Cofilin‐1 in human health. Recent studies reported the dysfunction of keratin related proteins in AD patients, such as loss function of keratin filament protein filaggrin^30^, ^31^ or downregulation of the tight junctions protein claudin‐1.^32^ However, few study concerns the role of actin dynamics in AD pathomechanism. Our research provides an insight among actin, cell mobility, skin barrier and AD. Moreover, large‐scale clinical research could be conducted in the future to validate if Cofilin‐1 can be a novel biomarker in AD patients. Additionally, the underlying molecular mechanism between Cofilin‐1 and AD progression need to be further investigated.

Due to the complex pathogenesis of AD, patients have different subtypes and significant heterogeneity.^33^ The prognosis of AD is affected by many factors such as chemical irritants, airborne allergens, food and microorganisms. Therefore, single biomarkers are difficult to accurately predict the occurrence, severity and outcome of the disease.^34^ At present, the clinical assessment of AD severity is primarily based on scoring atopic dermatitis (SCORAD), eczema area and severity index (EASI). It is hoped that future exploration in the field of AD‐related biomarkers can make breakthroughs in the direction of multi‐omics data integration, computer model construction, and large cohort clinical study validation, and the combination of SCORAD/EASI score and biomarker detection as the overall level measurement may be an important direction of subsequent research.

## AUTHOR CONTRIBUTIONS

X.Z. and J.Z. performed the experiments and data collection, B.X., L.Z. and Xiaobo L. performed the bioinformatic and statistical analysis, X.W. and Y.S. collected the clinical samples, S.Z. and H.Z. analyzed experimental data, Z.Z. and Xichuan.L. designed the study and wrote the manuscript. All authors contributed to the article and approved the submitted version.

## CONFLICT OF INTEREST

The authors declare no conflict of interest.

## Data Availability

The data that support the findings of this study are available on request from the corresponding author. The data are not publicly available due to privacy or ethical restrictions.
